# *Notes from the Field:* Interventions to Reduce Measles Virus Exposures in Outpatient Health Care Facilities — New York City, 2018

**DOI:** 10.15585/mmwr.mm6836a2

**Published:** 2019-09-13

**Authors:** Karen A. Alroy, Neil M. Vora, Robert J. Arciuolo, Mekete Asfaw, Beth M. Isaac, Martha Iwamoto, Antonine Jean, Denise H. Benkel, Kathleen Blaney, Bindy Crouch, Anita Geevarughese, Krishika A. Graham, Maura Lash, Demetre Daskalakis, Jane R. Zucker, Jennifer B. Rosen

**Affiliations:** ^1^Epidemic Intelligence Service, CDC; ^2^New York City Department of Health and Mental Hygiene, Long Island City, New York; ^3^Division of State and Local Readiness, Center for Preparedness and Response, CDC; ^4^Immunization Services Division, National Center for Immunization and Respiratory Diseases, CDC.

Strengthening health care facility infection control is crucial to preventing infectious disease transmission. Guidelines to prevent or minimize airborne pathogen spread in outpatient health care facilities exist (*1*); however, few reports describe practical implementation when engineering controls, such as recommended airborne infection isolation rooms (negative pressure rooms), are unavailable* (*2*). On September 30, 2018, a person with measles, a highly contagious respiratory illness characterized by fever and rash, that is spread by airborne transmission, was detected in New York City (NYC),^†^ and as of December 10, 42 laboratory or epidemiologically linked cases had been confirmed. By September 3, 2019, with 654 confirmed cases, this measles outbreak had become the largest in the United States since 1992, well before endemic domestic measles transmission was declared eliminated in 2000^§^^,^^¶^ (*3*,*4*). Interventions used in 15 outpatient health care facilities to attempt to prevent health care facility exposure from patients with suspected measles were evaluated.

During December 10–12, 2018, the NYC Department of Health and Mental Hygiene (DOHMH) surveyed the 17 NYC outpatient health care facilities that reported one or more suspected measles cases during September 30–December 10, 2018, to understand infection control procedures and share best practices. The facilities included seven group practices, four single-provider practices, four federally qualified health centers, and two urgent care centers. The primary staff member responsible for infection control at each facility was invited to participate in the survey.

Among the 17 contacted facilities, 15 participated. All 15 reported posting signs about measles symptoms and conducting patient screening for fever or rash. Thirteen facilities screened by telephone while scheduling appointments, 12 screened at check-in, and 10 screened both during scheduling and at check-in. Although no facility reported having a negative pressure room, 14 examined patients with suspected measles in a private exam room and prohibited subsequent use of that room for 2 hours.

Alternative isolation interventions were used by 13 facilities to attempt to minimize exposures from potentially infectious patients. These interventions included examining patients outdoors, including in their cars (10 facilities), having patients use separate entrances or examination spaces that were removed from the general patient population (six), evaluating patients after normal business hours (four), and conducting home visits (four).

When measles virus exposures occur in health care facilities, identifying and notifying nonimmune exposed persons and offering postexposure prophylaxis, when indicated, can be time- and human resource–intensive (*5*). Although most hospitals have infection control protocols that include use of negative pressure rooms, most outpatient facilities do not (*6*). No surveyed facility in this evaluation had a negative pressure room, the lack of which could make controlling airborne measles virus transmission in outpatient settings difficult (*6*). However, if they lacked a negative pressure room, most surveyed health care facilities used strategies to attempt to reduce exposures to measles and other airborne-transmitted pathogens, save time and human resources, and minimize health care–associated transmission of measles. Although this report does not assess the effectiveness of these interventions, it shares strategies to attempt to reduce measles exposures in health care facilities. The essential common element in the implemented strategies is early awareness that a patient might have measles, optimally before that patient enters the health care facility. This underscores the importance of maintaining a high index of suspicion during an outbreak, performing measles screening, and rapidly identifying patients with suspected measles.

## Footnotes

* https://www.cdc.gov/infectioncontrol/guidelines/isolation/index.html.

† https://www1.nyc.gov/assets/doh/downloads/pdf/han/alert/2018/alert38-measles-outbreak.pdf.

§ https://www1.nyc.gov/assets/doh/downloads/pdf/han/alert/2019/measles-transmission-ended.pdf.

¶ https://www.cdc.gov/measles/cases-outbreaks.html.
