# *Erratum:* Vol. 68, No. 36

**DOI:** 10.15585/mmwr.mm6838a5

**Published:** 2019-09-27

In the report “Notes from the Field: Interventions to Reduce Measles Virus Exposures in Outpatient Health Care Facilities — New York City, 2018,” on page 791, the Acknowledgments should have read “L. Hannah Gould, New York City Department of Health and Mental Hygiene; Mona Marin, Jennifer Wright, **Zeshan Chisty**, CDC; all staff members from participating health care facilities in New York City.”

